# Characterization and Molecular Profiling of *PSEN1* Familial Alzheimer's Disease iPSC-Derived Neural Progenitors

**DOI:** 10.1371/journal.pone.0084547

**Published:** 2014-01-08

**Authors:** Andrew A. Sproul, Samson Jacob, Deborah Pre, Soong Ho Kim, Michael W. Nestor, Miriam Navarro-Sobrino, Ismael Santa-Maria, Matthew Zimmer, Soline Aubry, John W. Steele, David J. Kahler, Alex Dranovsky, Ottavio Arancio, John F. Crary, Sam Gandy, Scott A. Noggle

**Affiliations:** 1 The New York Stem Cell Foundation, New York, New York, United States of America; 2 Department of Pathology & Cell Biology and the Taub Institute for Research on Alzheimer's Disease and the Aging Brain, Columbia University, New York, New York, United States of America; 3 Departments of Neurology and Psychiatry and the Alzheimer's Disease Research Center, Icahn School of Medicine at Mount Sinai, New York, New York, United States of America; 4 Department of Psychiatry, Columbia University, New York, New York, United States of America; 5 James J Peters Veterans Administration Medical Center, Bronx, New York, United States of America; University of Florida, United States of America

## Abstract

Presenilin 1 (*PSEN1*) encodes the catalytic subunit of γ-secretase, and *PSEN1* mutations are the most common cause of early onset familial Alzheimer's disease (FAD). In order to elucidate pathways downstream of *PSEN1*, we characterized neural progenitor cells (NPCs) derived from FAD mutant *PSEN1* subjects. Thus, we generated induced pluripotent stem cells (iPSCs) from affected and unaffected individuals from two families carrying *PSEN1* mutations. *PSEN1* mutant fibroblasts, and NPCs produced greater ratios of Aβ42 to Aβ40 relative to their control counterparts, with the elevated ratio even more apparent in *PSEN1* NPCs than in fibroblasts. Molecular profiling identified 14 genes differentially-regulated in *PSEN1* NPCs relative to control NPCs. Five of these targets showed differential expression in late onset AD/Intermediate AD pathology brains. Therefore, in our *PSEN1* iPSC model, we have reconstituted an essential feature in the molecular pathogenesis of FAD, increased generation of Aβ42/40, and have characterized novel expression changes.

## Introduction

Although the majority of Alzheimer's disease (AD) cases are late onset and likely result from a mixture of genetic predisposition and environmental factors, there are autosomal dominant genetic forms of the disease that affect patients at much earlier ages (FAD). Known familial early-onset genes include mutations in amyloid precursor protein (*APP*), presenilin-1 (*PSEN/PS1*), and presenilin-2 *(PSEN2/PS2*)[1]. *PSEN1* mutations are responsible for the most common form of inherited AD and are 100% penetrant [1]–[3]. The most prevalent theory for the underlying cause of AD is the “amyloid hypothesis”, in which toxic oligomerogenic forms of Aβ, a cleavage product of APP, accumulate and cause neuronal dysfunction and cell death [4]. PS1/PS2 are key components of the γ-secretase complex that mediates one of the two APP cleavage events, and mutations in *PS1* increase the relative ratios of the more oligomerogenic Aβ species (i.e. Aβ42) to less oligomerogenic species (Aβ40).

Most investigation of the molecular phenotypes caused by the *PSEN1* mutations has focused on this microheterogeneous cleavage at the carboxy terminus of Aβ. This qualitative change is believed to be associated with hypomorphism in processivity [5] and has implications for misprocessing of multiple substrates other than APP [6]. Further, the magnitude of the mutant *PSEN1*-associated perturbations of Aβ42:Aβ40 varies widely, and, in some mutations (e.g., *PSEN1 L271V* in the Tas-1 family;[7]) alterations in the Aβ42:Aβ40 ratio have been either minimal or difficult to demonstrate. This raises the possibility that PS1 could have physiological or pathological effects independent of its effects on APP processing. This is an important issue to investigate thoroughly since *PSEN1* mutations are present in virtually all of the cell- and mouse-based models used to develop hypotheses and treatments for common, sporadic AD. However, in common, sporadic AD, no *PSEN1* mutation is present. Indeed, *PSEN1*-mutation-related AD is conceived as a disease of Aβ *anabolism* while at least some forms of common, sporadic AD (i.e., that linked to *APOE4*;[8]) are conceived as a disease of Aβ *catabolism*. Other genes linked to common, sporadic AD (e.g., CR1) appear to act via the immune response and may modulate cerebral amyloidosis in unexpected ways [9].

Recently several groups have generated human iPSC or transdifferentiation models of AD, with studies primarily focused on FAD neurons [10]–[13]. None of these studies addressed whether there are any differences between AD and control NPCs prior to neuronal differentiation. NPCs are a potentially relevant system to study aspects of disease on neuronal differentiation. Some FAD mouse models demonstrate deficits in neurogenesis as the animals age, and NPCs taken from AD brains of recently deceased patients have decreased neurogenic potential in comparison to those from similarly aged healthy controls [14], [15]. Newly born adult neurons in mouse models of AD have also been reported to have significantly decreased viability relative to control mice [16]. In addition, the brains of early-onset Alzheimer's patients might have developmental alterations that could affect the progression of the disease. This possibility has been recently speculated in response to a report that young adults from the Colombian FAD kindred (PS1 E280A) have changes in grey matter and synaptic function potentially prior to formation of Aβ plaques [17](http://dx.doi.org/10.1016/S1474-4422(12)70256-9). NPCs are also a more homogenous population that might reduce the experimental variability of mature neurons produced by current neuronal differentiation protocols, and thus could be a better system to identify novel molecules potentially important for early events in AD. We used gene expression profiling (GEP) of this population to identify novel candidate genes and confirmed hits in brains from common, sporadic AD with advanced or intermediate pathology by qPCR and by comparison to published transcriptomes of laser captured microdissected (LCM) cortical neurons from brains with AD pathology.

## Results

### Generation of iPSC Lines

**Figure 1:**
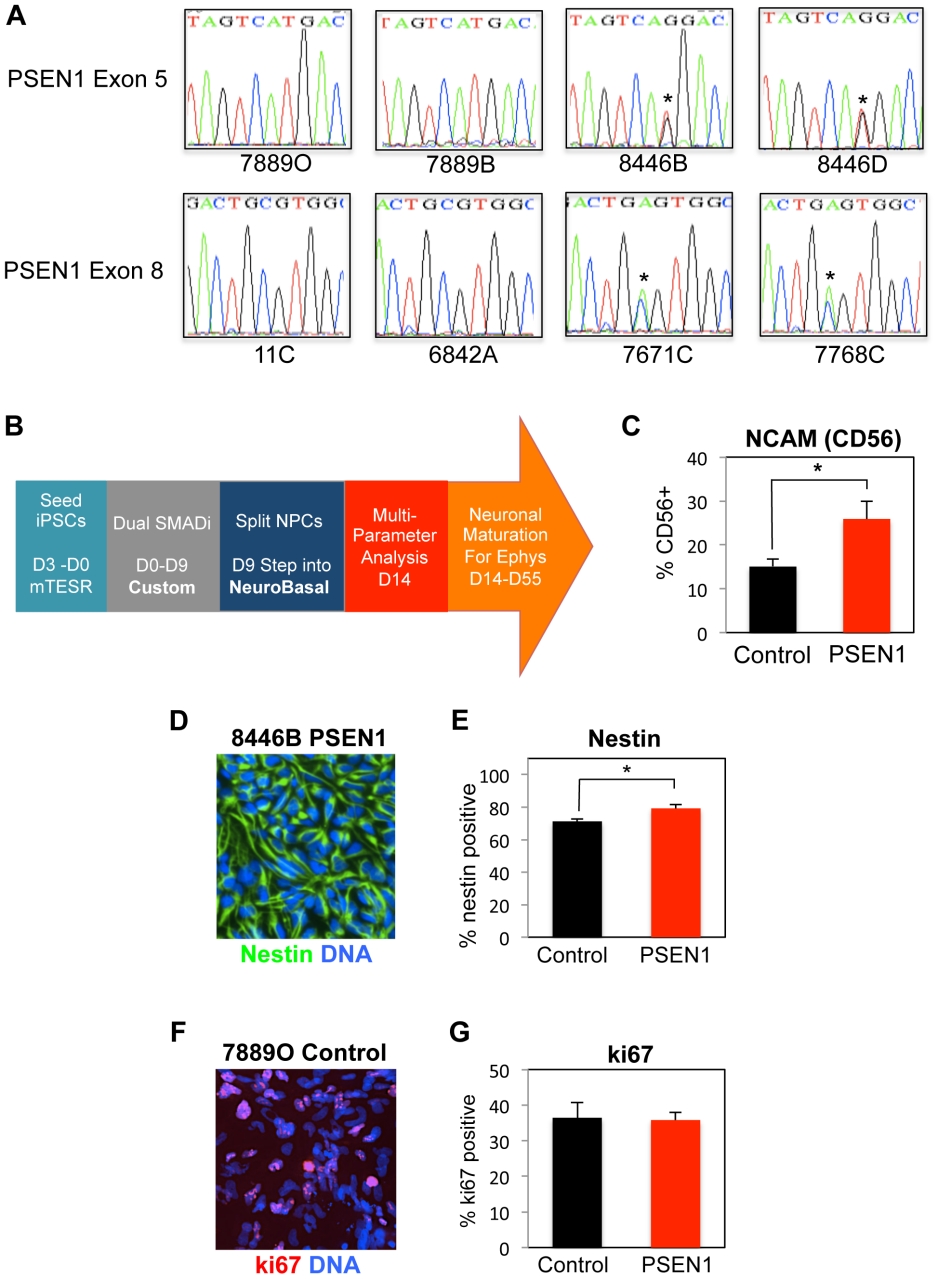
iPSC Characterization and Neuronal Differentiation. *A*. Sequencing of core set iPSCs for *PSEN1* mutations in exon 5 (M146L) and exon 8 (A246E) respectively. * marks site of the mutations. *B*. Cartoon of neuronal differentiation scheme, showing timing and changes into different medias as described in Fig S6. *C.* All 8 core lines were neuronally differentiated for 14 days and were analyzed by flow cytometry for the expression of CD56 (NCAM). The aggregate result of three independent experiments is shown. *PSEN1* cells have a small but statistically significant increase in NCAM+ surface expression (control vs. PSEN1, n = 12 for each genotype, p = 0.02, Student's t-Test, error bars reflect SEM). *D*. Representative immunostaining for the neural progenitor marker nestin in iPSC line 8446B. Nestin is in green, DNA is in blue. *E*. Quantification of nestin staining for aggregate data from two independent experiment, with 3 biological replicates for the 8 core lines in each experiment. For control vs. PSEN1, n = 8 for each genotype, p = 0.02 (Student's t-Test, error bars reflect SEM). *F*. Representative immunostaining for the cell cycle marker Ki67 in cell line 7889O. ki67 is in green, DNA is in blue. *G*. Quantification of ki67 staining for aggregate data from three independent experiments, with 3 biological replicates for the 8 core lines in each experiment. See also Fig. S1.

In order to create *PSEN1* mutant and wild-type control iPSC lines, established fibroblast lines were obtained from the cell bank repository at the Coriell Institute (Camden, NJ). Non-EBV transformed fibroblast lines were selected from the “Canadian” (FAD1, A246E PS1 mutation) and the “Italian” (FAD4, M146L PS1 mutation) EOFAD kindreds. Heterozygosity in the *PSEN1* locus was confirmed in AD patients for fibroblasts (data not shown) and subsequently derived iPSCs via sequencing (Fig 1A). Fibroblast lines were reprogrammed using four high-titer retroviral constructs prepared by the Harvard Gene Therapy Core Facility that encoded human Oct4, KLF4, SOX2 and c-Myc, respectively [18]. iPSC colonies were initially selected by morphology, passaged several times to remove transformed cells, and expanded before characterization.

### Characterization of iPSCs

After iPSCs were expanded to multi-well format, they were characterized using a variety of quality control assays. Initial characterization included the presence of alkaline-phosphatase (AP) enzymatic activity, immunostaining for pluripotency markers, and qPCR for both endogenous pluripotent markers and viral transgene silencing. An example of initial characterization of one line (7768C) is shown in Fig. S1. Cell lines with insufficient transgene silencing were not further analyzed.

### Selection and Further Characterization of Core Set of iPSC Lines

We selected 8 iPSC lines, including one unrelated control iPSC line 11C [19] to serve as a core set for the majority of our experiments (Table 1, Fig. S1, S2). All data utilizing the core set shows the same order of cell lines as in Table 1. The best transgene shutoff and endogenous expression of stem cell genes were used as the main criteria in clone selection. In addition, core set candidates were also karyotyped (e.g. Fig S1C) and fingerprinted (Cell Line Genetics; data not shown) to ensure that they matched the parental fibroblast line. Unfortunately, five iPSC clones from two patients from the FAD1 family harbored chromosomal mutations of various types (data not shown). Since we were unable to obtain a karyotypically normal iPSC line from the FAD1 family, we decided to use clone 6842A that had a balanced translocation, t(17(q22.3),19(q13.4)). Many individuals harbor balanced translocations without issue [20]. In addition, for one FAD4 control individual (7889) and one FAD4 AD patient (8446), we selected two clones for further study, in order to test for the possible effects of random transgene insertion. Determination that iPSC clones from each patient were independent as defined by different viral integration sites was determined by Southern blotting (Fig S2). Integration events were analyzed for two different transgenes: Oct4 and Klf4.

**Table 1:** Core set of iPSC Lines.

Line	Family	Sex	Age	PSEN1	APOE
7889O	FAD4	M	18	WT	ε3/3
7889B	"	"	"	"	"
11C	N/A	M	36	WT	ε3/ε3
6842A	FAD1	M	75	WT	ε3/ε3
8446B	FAD4	M	38	M146L	ε3/ε3
8446D	"	"	"	"	"
7671C	FAD1	M	44	A246E	ε3/ε3
7768C	FAD1	F	31	A246E	ε3/ε4

See also Fig S2.

*In vitro* pluripotency of core lines was demonstrated by undirected differentiation of iPSCs into embryoid bodies and subsequent immunostaining of frozen sections for germ-layer specific markers for each of the three developmental germ layers (Fig S2). For cell line 7768C, we also established *in vivo* pluripotency via subcutaneous injection of undifferentiated iPSCs within a matrigel matrix into the dorsal flank of NSG immune-compromised mice (Jackson Laboratory). As shown in Fig S1, the ability to form three germ layers was assessed using hematoxylin and eosin (H&E) staining of paraffin-embedded sections of resulting teratomas. Observed tissues include: glandular epithelia, indicating the presence of endoderm; bone and cartilage, indicating differentiation of mesoderm lineages; and neural epithelia, including areas with retinal pigmented epithelium, indicating competence of differentiation towards ectodermal lineages.

### Analysis of *APOE* Genotype

*APOE* isotype is the most common identified risk factor associated with late-onset AD and could potentially influence observed phenotypes [1]. *APOE* genotype was determined by standard restriction fragment length polymorphism method [21]. 7 of 8 lines in our core set are *APOE* ε3/ε3, the reference genotype (Table 1). One line, 7768C is *APOE* ε3/ε4, and thus harbors both one PS1 A246E early-onset familial AD deterministic allele and one late-onset AD risk allele.

### Neuronal Differentiation of iPSC Lines

In order to investigate *PSEN1* and control NPCs, we differentiated cells by a monolayer method and analyzed cells at different time points during this process. As shown in Fig 1B in cartoon form, iPSCs were plated as single cells, allowed to recover, and subsequently neuralized by inhibition of both branches of TGFβ signaling pathways (dual-SMAD inhibition, also see Fig S6)[22]. Inhibition of TGFβ pathways is sufficient to induce anterior neural fates from pluripotent cells [23]. To look at a mixture of mostly NPCs and a minority of early-born neurons, we assessed our cell lines at Day 14 post dual-SMAD inhibition. There was a significant difference in the amount of CD56+ (NCAM) *PSEN1* cells as compared to control cells as measured by flow cytometry (FCM) of live cells as determined by forward scatter plot (26% vs. 15%, p = 0.02, Student's t-Test, Fig 1C). Although CD56+ expression is often used to identify neuronal populations by FCM, it is also expressed in some nestin-positive progenitors [24]. At day 14 of neuronal differentiation there were no NeuN+ cells, a more mature neuronal marker (data not shown). However, there were small patches of Tuj1+ cells with more complex neurite morphology, which appeared to be more prevalent on average in PSEN1 cells (Fig S1).

The largest proportion of cells at this time point was nestin-positive NPCs (Fig 1D, E). *PSEN1* lines had a small but statistically significant increase in the percentage of nestin-positive cells compared to control lines (79% vs. 71%, p = 0.012, Student's t-Test). A significant proportion of cells were in cell cycle as measured by Ki67 staining (Fig 1F, G; average for 8 lines: 33%), which was not statistically different between PSEN1 genotypes.

### NPCs Have the Capacity to Make Electrically-Active Neurons

**Figure 2:**
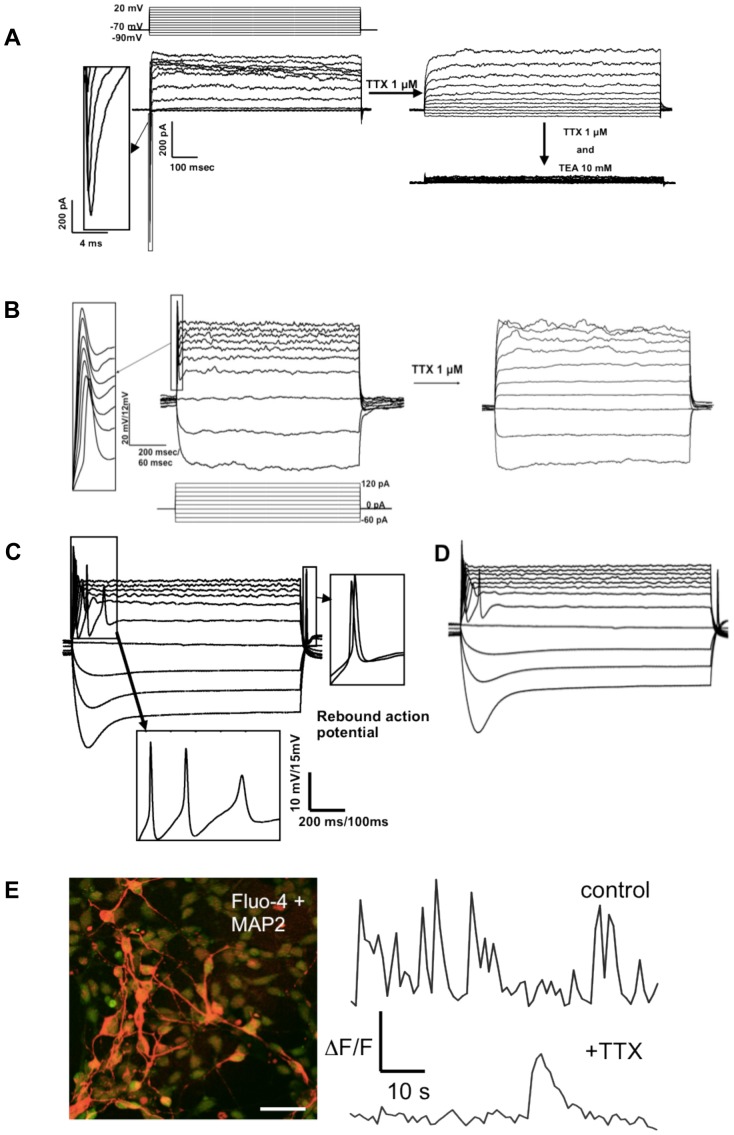
Action potentials and normal spontaneous Ca2+ transients are present in neurons differentiated from control line 7889O and 8446. *A.* Representative traces in voltage clamp mode showing fast inward currents followed by long-lasting outward currents. Voltage 10 mV steps are shown in the upper panel. The inset shows an enlarged view of the inward current. (7899O, day 55). Inward sodium currents and potassium currents were observed on 13 out of the 22 cells analyzed at this time-point. The average Resting Membrane Potential (RMP) was equal to −45.94 mV±2.77 (standard error, s.e.; *n* = 17). Following initial recording cells were perfused with 1 µM TTX (tetrodotoxin) to block sodium currents, and subsequently with 10 mM TEA (tetraethylammonium) to block potassium currents. *B*. Representative action potentials in response to step current injections of 20 pA (lower panel) in current clamp mode (7889O, day 45). The average RMP was equal *to −37.93 mV*±*6.16 (n = 13).* Action potentials were observed on 4 out of 10 cells analyzed at this time point. Following initial recording the action potential was blocked by perfusing cells with 1 µM TTX. *C*. Representative action potentials in response to step current injections of 20 pA as in B in current clamp mode (7889O, day 55). They show trains of action potentials upon depolarizing current injection and “rebound” action potentials at the end of hyperpolarizing current injections. Action potentials were observed on 13 out of the 22 cells recorded at this timepoint. Out of them, 3 had a rebound action potential like the one shown on the inset of this panel. *D*. Representative action potentials in response to step current injections of 20 pA from 8446B, day 55. Trains of action potentials upon depolarizing current injection and “rebound” action potentials at the end of hyperpolarizing current injections are visible. Action potentials from 8446 cell line were observed on 8 out of the 15 cells recorded at this timepoint. *E*. (A) Image of 7889O loaded with Fluo-4NW (green) and stained for MAP2 (red) after Ca2^+^ transients have been recorded. (B) Representative spontaneous Ca2^+^ spikes recorded from 7889O neurons before and after application of TTX (N = 54 for control, N = 22 for TTX). Spiking frequency was significantly slower after application of TTX and the kinetics of each individual spike was slower, suggesting that 7889O cells display normal neuronal Ca2+ transients.

We wished to establish that our NPCs were capable of making mature neurons that had electrical signaling properties similar to primary neurons. Thus, we recorded electrophysiogical properties from 375 cells that had been neuronally differentiated 35 to 55 days from control iPSC line 7889O and 87 cells from PS1 iPSC line 8446B. Cells differentiated into mature neurons as shown by the presence of fast inward currents due to the Na^+^ channels opening after depolarization with a series of 10 mV voltage steps from −90 mV (Fig 2A) and their ability to produce action potentials upon stimulation with a depolarizing current, as shown for 7889O (Fig 2B,C) and 8446B (Fig 2D).

**Figure 3:**
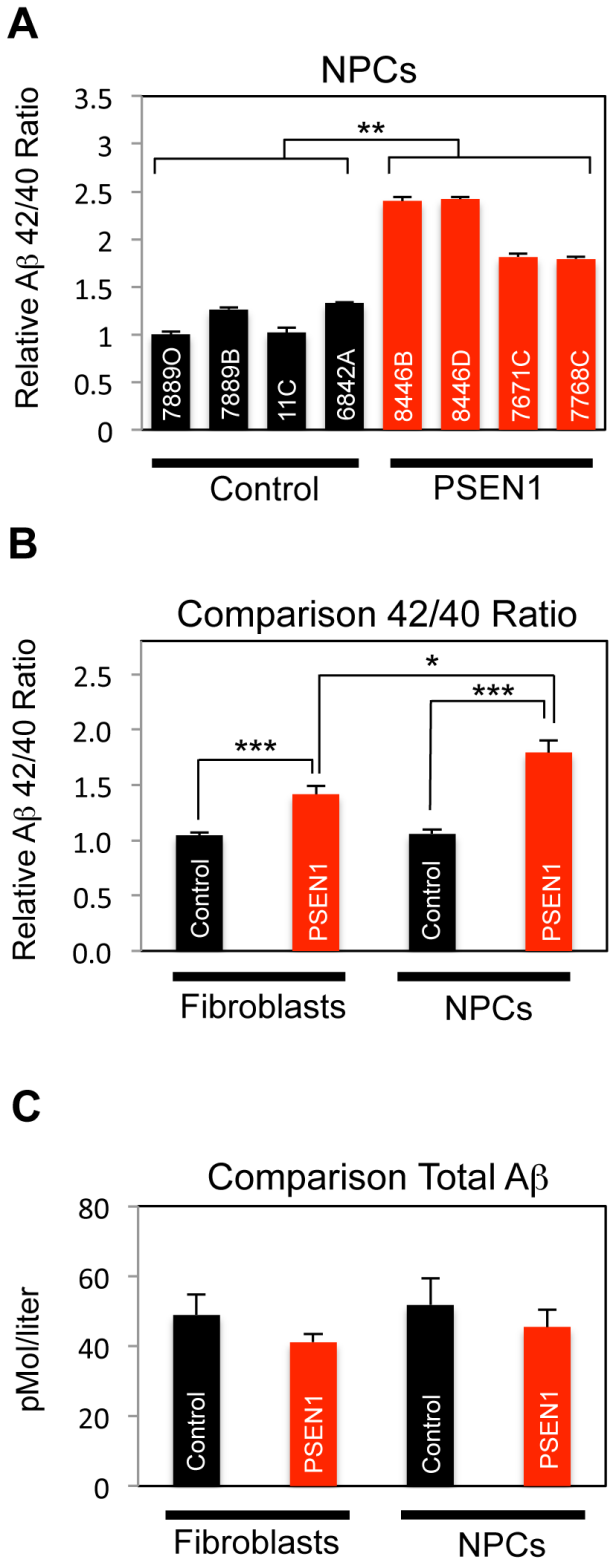
Aβ42/Aβ40 Ratio is Elevated in *PSEN1* Cells. All assays detected Aβ1-40 and 1-42 using ELISA (Wako) on conditioned media from the cell type indicated. Ratios were normalized against the first control line listed on each panel. Statistical significance was determined via Student's t-Test, error bars reflect SEM. Each n equals an individual cell line (averaged biological triplicates) in 1 independent experiment. *A*. Aβ42/Aβ40 ratio is increased in day 14 differentiated NPCs/early neurons. Control and *PSEN1* NPCs were generated from the core set of iPSC lines, and one of three independent experiments is shown. For control compared to PSEN1 NPCs (n = 4 for each genotype), p = 0.003. *B–C.* Aggregate data is shown from 3 independent fibroblast and 3 independent NPCs/early neuron experiments. N = 7 for each fibroblast genotype data point, and n = 12 for each NPC/early neuron genotype data point. *B*. Aβ 42/40 ratios are shown for both control and PSEN1 fibroblasts and NPCs. For control fibroblasts vs. *PSEN*1 fibroblast, p = 0.001; for control NPCs vs. *PSEN1* NPCs, p = 0.000005; for *PSEN1* fibroblasts vs. *PSEN1* NPCs, p = 0.036. *C*. Total Aβlevels (Aβ40 + Aβ42) are statistically similar between control and *PSEN1* fibroblasts and NPCs/early neurons. See also Figure S3.

As further evidence of maturation, 7889O cells exhibited a single action potential following depolarizing current injection at 45 days, while at 55 days they responded with a repetitive AP firing pattern and a rebound action potential after a hyperpolarizing current pulse (Fig 2C). A similar evolution was observed with 8446B cells, as shown by the presence of multiple action potentials in Fig 3D. Both inward currents and action potentials were blocked by perfusion with 1 µM tetrodotoxin (TTX, Fig 2A,B), indicating that the currents were due to activation of Na^+^ channels. Furthermore, the K^+^ channel blocker tetraethylammonium (TEA) cancelled the long lasting outward current after the fast inward current, suggesting the presence of K+ channels. At Day 55, 60% of cells were capable of firing trains of action potentials, and 13% had “rebound” action potentials at the end of hyperpolarizing current injections (Fig 2B,C).

We also assessed electrical activity by looking at Ca^2+^ activity. Ca^2+^ transients are used by neurons to regulate cellular homeostasis by modulating activity-dependent gene expression, controlling neurotransmitter release, and regulating membrane excitability [25]. Therefore, we asked whether our neurons displayed normal Ca^2+^ transients that were sensitive to TTX. To measure cytosolic Ca^2+^, we preloaded neuronally differentiated (45 days) iPSC line 7889O with Fluo-4NW and recorded spontaneous Ca^2+^ spikes during a two-minute interval (Fig 2E). We selected a 500 µM-by-500 µM imaging area in each dish of cells (N = 5 dishes), which yielded an average of 34.5 cells per imaging window. Of these cells, 13.2 cells, or approximately 40% rendered measureable Ca^2+^ spikes. We observed a spike frequency of about 0.8 Hz, with an average inter-event interval of 0.045 Hz, approximating what has been observed in both cultured cortical neurons and neurons derived from human iPSCs [26]. After application of 1 uM TTX for 5 minutes, the number of cells that displayed measurable spontaneous Ca^2+^ activity decreased to about 19%, and of those, only a few demonstrated at least one event during the 2-minute imaging time-window. Of the few cells that did show a Ca^2+^ transient, the kinetics were generally slower, again mirroring what has been observed in cultured cortical neurons [27].

### Aβ Production

After establishing the authenticity of our functional NPC model, we next wished to investigate protein processing associated with PS1 function and dysfunction. According to the amyloid hypothesis, oligomerized Aβpeptides are responsible for aberrant synaptic plasticity and cellular toxicity [4]. Fibroblasts from *PSEN1* mutant patients have been observed to produce an increased ratio of Aβ42/Aβ40, thus enhancing the relative levels of the more oligomerogenic Aβ peptide [28], [29]. In congruence with these earlier studies, we also observed an increase in the ratio of Aβ42/Aβ40 secreted by human fibroblasts via analysis of conditioned media by ELISA (data not shown and Fig 3B). We then looked at Aβproduction in NPCs/early neurons (14-day differentiation) where this parameter has not been previously assessed. The Aβ42/Aβ40 ratio was also increased in conditioned media from *PSEN1* NPCs and early neurons as compared to control lines (Fig 3A). Aβ42/Aβ40 ratio between *PSEN1* and control cells appeared to increase in magnitude following neuronal differentiation (Fig 3B), similar to what has been reported for transdifferentiated *PSEN1* neurons [12]. The amount of total Aβ produced from control and *PSEN1* fibroblasts or NPCs were statistically equivalent (Fig 3C), indicating that APP processing is altered in *PSEN1* cells in terms of quality rather than quantity. Many mutant PS1 proteins have been shown to be hypomorphic [30]; i.e., the total number of moles of both Aβspecies generated per mole of APP catabolized. Similar to what has been found in numerous studies in other cell types [31], a γ-secretase inhibitor (10 µM DAPT) was sufficient to block Aβproduction in both control and PS1 cells (Fig S3).

### Gene Expression Profiling (GEP) Comparison for Control Versus *PS1* NPCs

Having established that we could successfully model the molecular pathology of FAD in our *PSEN1* NPCs, we further defined their gene expression via global gene expression studies. The purpose of this approach was threefold: 1) Further characterization of *PSEN1* NPCs, including additional analysis of their neurogenic potential; 2) Identification of molecules that might have a developmental and/or amyloid-independent role in the pathogenesis of FAD; 3) Attempt to find molecules that might also be misregulated in late-onset AD. Thus, we performed GEP experiments on both undifferentiated and 14-day neuronally differentiated control and *PSEN1* NPCs using the Illumina HumanHT-12-14 BeadChip platform. The results were analyzed using Genome Studio software, and genes were considered differentially expressed if DiffScores were greater than 13 or less than -13 (p = 0.05).

**Figure 4:**
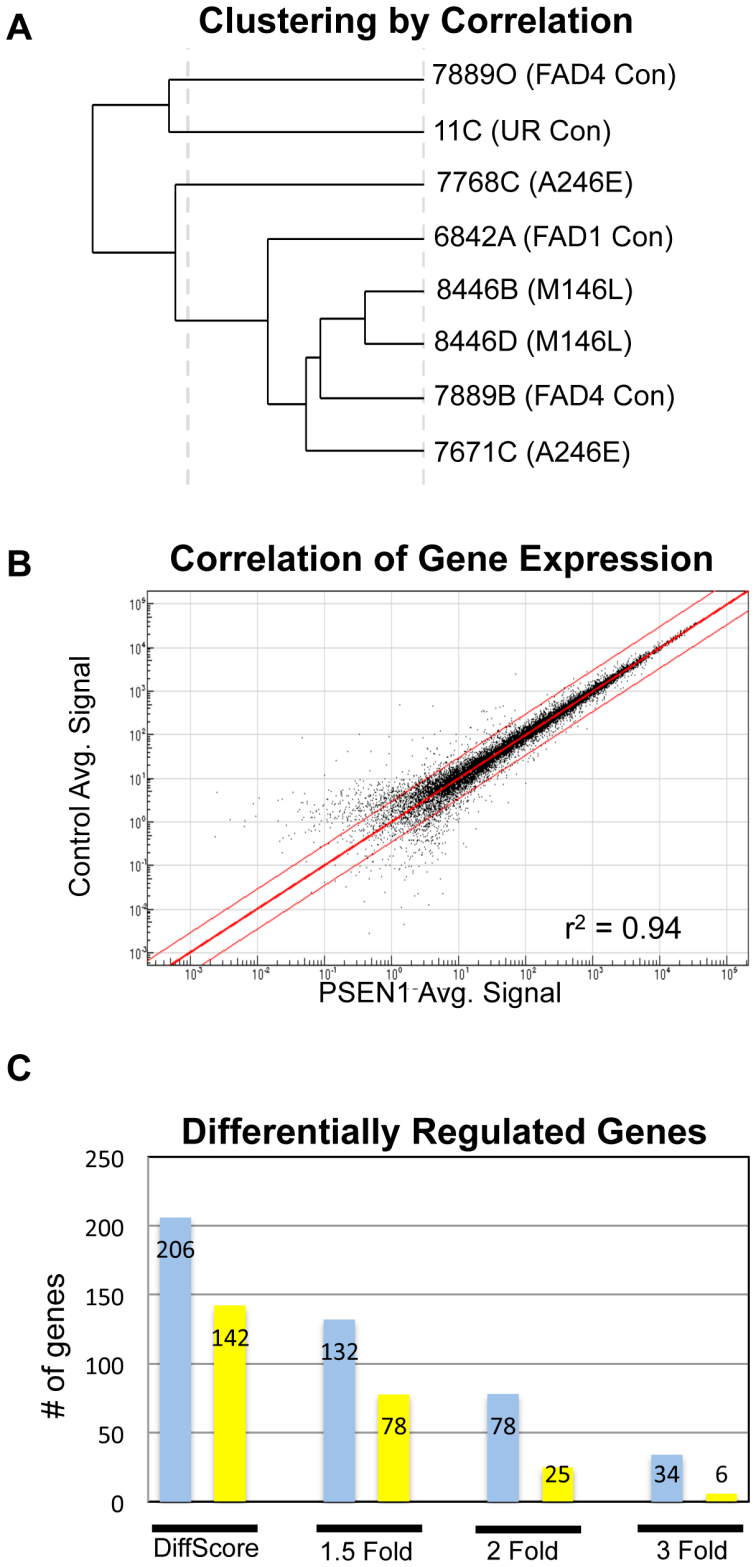
Gene Expression Profiling of Control vs. PS1 NPCs/Early Neurons. All 8 core iPSC lines were differentiated for 14 days in triplicate wells, lysed for RNA, amplified to generate cRNA, and ran on the Illumina HumanHT-12-14 BeadChip platform. *A*. Clustering of 8 core lines by correlation. UR stands for unrelated control. *B*. Scatter plot (log scale) of the correlation of gene expression between 4 control lines and 4 PSEN1 lines. The red lines indicate a 3-fold expression difference. *C*. Chart indicating the number of upregulated (shown in blue) and downregulated (shown in yellow) genes for each threshold of analysis. “DiffScore” refers to genes with a Diff Score of >13 (upregulated) or <13 (downregulated), which indicate a change in expression with a pValue of p≤0.05, without regard to the relative fold change. Criteria for fold change categories include the listed fold change as well as statistical significance. See also Table S1.

For undifferentiated iPSCs, six lines were used: 7889O, 11C, 6842A, 8446B, 7671C, and 7768C (representing all six patients in our core set; 1 sample per line). There was only one differentially expressed gene between 3 control and 3 *PSEN1* lines, *NLRP2*. However, after neuronal differentiation into NPCs there was a significant increase in gene expression differences. RNA from all 8 core lines was amplified and run in triplicate. 22 of 24 samples were independent biological replicates, while 2 were technical replicates. Control and PS1 cells did not segregate by genotype (Fig 4A) and when 4 control lines were compared to 4 *PSEN1* lines, the majority of genes have overall similar expression as shown by scatter plot (Fig 4B, correlation coefficient 0.94). Importantly, FAD1 and FAD4 family members also did not segregate by family. This suggests that related controls might not be more beneficial than unrelated controls, although sibling or parental controls might prove more advantageous than the more distant relationships used in this study.

Despite the overall similarity in expression between control and *PSEN1* cells, and the lack of clustering by genotype, utilizing the DiffScore parameters described above, there were 206 upregulated genes and 142 downregulated genes in *PSEN1* cells relative to control NPC cultures (Fig 4C). We used DAVID Functional Annotation on each list to identify relevant gene ontology (GO) terms (Table S1) [32], [33]. Using a threshold minimum of 10 genes per GO term, we were able to identify 9 GO terms associated with upregulated genes and 20 GO terms associated with downregulated genes. While some of these terms have overlapping functions, some striking examples include an increase in genes associated with inhibition of gene transcription and a downregulation of apoptosis-related genes (with a notable exception, *BIK*). Importantly, GO categories associated with neuronal function did not appear at this threshold of analysis. Utilizing the DAVID Functional Clustering Tool with parameters allowing smaller sets of genes, we were able to detect several neuronal GO term categories with 4–5 overlapping genes: *GFRA3*, *ISL1*, *DLX1*, *SEMA3B*, and *ERBB3*. Thus, while this supports a subtle increase in neurogenic potential for *PSEN1* NPCs, which would be consistent with our observation of a small increase in CD56+ surface expression (Fig 2C), the overall lack of substantial neuronal GO categories suggests that gene expression differences between *PSEN1* and control NPCs are not skewed by the minority of early-born neurons present at day 14 of differentiation.

### qPCR Validation of *PSEN1* NPC Differentially-Regulated Genes

We postulated that most positively and negatively regulated genes in our GEP experiments would be the most potentially relevant to AD. There were 34 upregulated genes (>3-fold increase, Diff Score >13) and 6 downregulated genes (>3-fold elevation in controls, Diff Score <−13) that were attractive candidates for further validation. We further pruned the upregulated list to 23 genes by eliminating 11 genes that were not expressed in control NPCs at statistically detectable levels. This reduced the potential of a rare minority cell type skewing the data.

We analyzed the remaining 29 genes in two independent differentiation experiments utilizing our 8 core cell lines in biological triplicates for each experiment (Table S2). The average expression of each of the four controls lines (n = 8, 2 experiments combined) was compared against the average expression of the four *PSEN1* lines (n = 8, 2 experiments combined). Ten upregulated genes (*ASB9*, *BIK*, *C7orf16*, *NDP*, *NLRP2*, *PLP1*, *SLC45A2*, *TBX2*, *TUBB4*, *ZNF300*) and four downregulated genes (*ADM2*, *FLJ35024*, *MT2A*, *PTGS2*) were validated by this method (Student's t-Test p<0.05). Four additional genes showed an upregulation trend in *PSEN1* NPCs (*ABCC2*, *ECEL1*, *EGFL8*, *FSTL5*, *SMOC1*). We then looked at the expression of three targets in more detail: *NLRP2*, *ASB9* and *NDP*.

### NLRP2, ASB9, and NDP Expression in NPCs

*NLRP2* (*NALP2*) was the only gene differentially regulated in undifferentiated iPSC GEPs. It is also one of the 10 *PSEN1* upregulated genes found in our NPC analysis. NLRP2 is a component of the inflammasome, a protein complex that activates pro-inflammatory caspases such as caspase-1 [34], [35]. As inflammation has been argued to play a critical role in AD [36], it is intriguing that a major modulator of inflammatory signaling might be different in FAD patients from birth. This becomes particularly interesting in the recent report where crossing of an FAD mouse model with either NLRP3 or caspase-1 null animals caused decreased Aβ accumulation and significant attenuation of synaptic and memory deficits [37].

**Figure 5:**
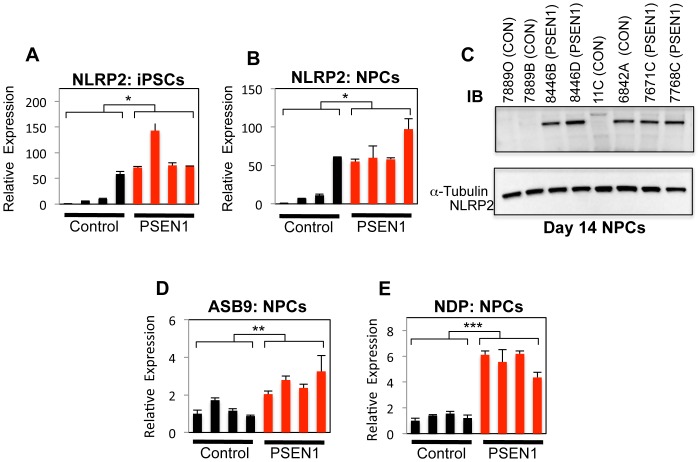
Validation of Target Genes in *PSEN1* NPCs. All qPCR data was normalized internally to *GAPDH* expression and also to cell line 7889O. Statistical significance was determined by Student's t-Test and error bars reflect SEM. *A*–*B*. *NLRP2* mRNA expression was assessed in undifferentiated iPSCs (*control vs. PSEN1*, n = 4 for each genotype, *p = 0.016*) and NPCs (*control vs.PSEN1, n = 4 for each genotype, p = 0.03*). *C*. Western blot analysis of NLRP2 protein expression in NPCs. α-Tubulin was used as a loading control *D*. Representative experiment showing *ASB9* mRNA expression in NPCs. For control vs. PSEN1, n = 4 for each genotype, p = 0.03. *E*. Representative experiment showing *NDP* mRNA expression in NPCs. For control vs. PSEN1, n = 4 for each genotype, p = 0.005. See also Table S2.

We first confirmed that *NLRP2* expression was upregulated in undifferentiated *PSEN1* cells. As shown in Fig 5A, *NLRP2* was expressed at much higher levels in the 4 *PSEN1* iPSC lines as well as control iPSC line 6842A (average 15-fold higher for those 5 lines compared to the other 3 control lines). Similar results were confirmed in *PSEN1* NPCs and 6842A at the mRNA and protein levels (Fig 5B,C). Interestingly, *NLRP2* is located on chromosome 19 at the breakpoint of the balanced translocation (q13.42) present in iPSC line 6842A.

ASB9 is a broadly expressed E3-ligase that targets creatine kinase B (CKB) and ubiquitous mitochondrial creatine kinase for degradation, and stable overexpression of ASB9 reduces mitochondrial membrane potential and affects mitochondrial morphology [38], [39]. CK activity has been shown to be lower in AD brains, and creatine has shown to be neuroprotective and is in clinical trials for multiple neurodegenerative diseases [40]. We confirmed ASB9 is upregulated over 2.4-fold in PS1 NPCs (Fig 5D). We were not able to detect ASB9 protein in our cells by immunostaining or Western blot (data not shown). It is perhaps not surprising that it might be expressed at low levels due to its deleterious effects on mitochondrial function.

Mutations in Norrie Disease Pseudoglioma (*NDP*, protein called Norrin) are responsible for Norrie disease, an X-linked recessive disorder, as well as several other rare eye disorders [45]. The primary manifestation of Norrie Disease is blindness and, in many cases, progressive hearing loss. In addition, in 30% or more of patients, mental retardation is also present, suggesting an important CNS role for NDP. Thus, NDP became an interesting target for further study, particularly in light of the hypothesis that FAD might have a developmental component. We confirmed by qPCR that NDP is in fact upregulated in *PSEN1* NPCs (Fig 5E). The aggregated data from three independent experiments indicated that NDP was upregulated 5.5 fold in *PSEN1* NPCs (p = 0.0002, Student's t-Test).

### Examination of *PSEN1* NPC Differentially-Regulated Genes in Late Onset AD Brains

We next assessed if the differentially-expressed genes in *PSEN1* NPCs could be reflected in brains from AD patients. We only analyzed late-onset AD brains as we were unable obtain brain tissue from FAD individuals. In addition, while our iPSC-derived NPCs may more closely resemble embryonic NPCs or adult NPCs rather than mature neurons, we hypothesized that some of our NPC hits might be altered in late-onset AD neuronal populations.

**Figure 6:**
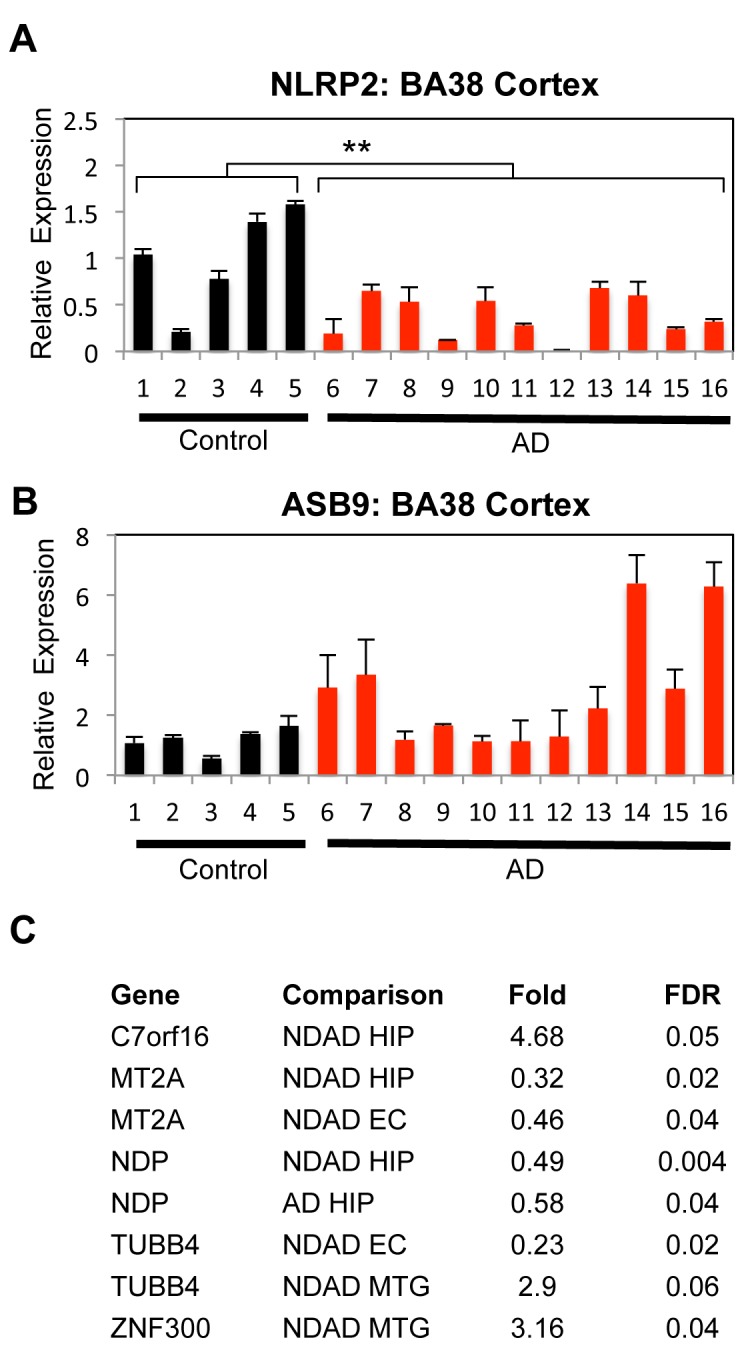
Examination of Target Genes in Late-Onset AD Brains. *A–B*. qPCR of NLRP2 (*A*) and *ASB9* (*B*) from mRNA from Brodmann's area (BA38) from control and AD brains. Black bars (1–5) are controls and red bars represent AD patients (6–16), which are described in Fig S5. qPCR data was normalized internally to *GAPDH* expression and also to the average of 5 control lines. Statistical significance was determined by Student's t-Test and error bars reflect SEM. For control vs. AD, n = 5 for control, n = 11 for AD, p = 0.005. *C*. List of *PSEN1* NPC target genes (Table S2) that have differential expression in independent microarray data of laser captured microdissected (LCM) cortical neurons from one of three brain areas (details in Fig S5). All comparisons are either non-demented AD pathology (NDAD) or AD versus control samples. HIP refers to hippocampus, EC for entorhinal cortex, and MTG for middle temporal gyrus. Fold change and significance (FDR: false discover rate) reflect values for LCM neuron arrays. See also Figure S4.

The first approach was to analyze *NLRP2*, *ASB9*, and *NDP* expression in AD brains by qPCR. mRNA isolated from Broadmann Area 38 (BA38, temporal pole) of 11 AD and 5 control patients was converted to cDNA and analyzed by qPCR for *NLRP2*, *ASB9* and *NDP*. The temporal lobe (including the temporal pole) is vulnerable in Alzheimer's disease, particularly in intermediate stages of the disease [41]. Interestingly, *NLRP2* expression was found to be statistically reduced in BA38 of AD patients, the opposite of our NPC results (38% of levels of controls, p = 0.005, Student's t-Test, Fig 6A). *ASB9* was elevated in BA38 of some AD individuals, but was only a trend when looking at all 11 AD patients compared to controls (Fig 6B). *NDP* expression was similar between control and AD patients by qPCR, but intriguingly was present in some neurofibrillary tangles in AD forebrains (Fig S4).

To further confirm the authenticity of hits, we analyzed publically available GEPs from laser capture microdissected (LCM) cortical neurons from control subjects or from patients with moderate or severe AD pathology [42], [43]. Some individuals displayed intermediate pathology individuals but without sufficient clinical criteria to be labeled as having AD; these cases were designated “non-demented individuals demonstrating AD pathology” (NDAD). We used our list of 14 differentially expressed genes to interrogate GEPs of hippocampus, entorhinal cortex (EC), and the middle temporal gyrus (MTG) from NDAD individuals and hippocampus from AD patients. Gene expression from these regions had already been compared to brain regions from age-matched control patients (Fig S6), and thus one could determine whether the genes misregulated in mutant *PSEN1* NPCs were similarly misregulated in early stages of AD pathology in vulnerable brain areas.

Five target genes had statistically altered expression in AD/AD pathology brains (Fig 6C). *C7orf16* (PPP1R17) and *ZNF300*, which are elevated in *PSEN1* NPCs, were also found to be more highly expressed in NDAD hippocampi and NDAD MTG respectively. One caveat is that *C7orf16* was expressed at very low levels. *MT2A*, which has reduced expression in *PSEN1* NPCs, was also expressed at significantly lower levels in NDAD hippocampi and EC. Interestingly, two other metallothionein proteins, *MT1A* and *MT1F*, were also significantly reduced in our GEP of *PSEN1* NPCs. Some studies report that MTs may attenuate Aβ toxicity [44]. On the other hand, *NDP*, which is elevated in *PSEN1* NPCs, is significantly lower in both NDAD and AD hippocampi. The situation with *TUBB4* (elevated in *PSEN1* NPCs) is more complex, as it is lower in NDAD EC, but increased as a trend in NDAD MTG. There was a trend toward upregulation of *ASB9*, although this did not reach statistical significance. While *NLRP2* itself was not differentially regulated in the brain regions analyzed, its homologue *NLRP1* was downregulated in MTG of brains designated NDAD (37%, p = 0.00005). Overall, our data support the hypothesis that at least some of our hits of genes differentially regulated in FAD NPCs are also differentially regulated in AD brains.

### Recombinant Norrin promotes primary neurosphere formation in adult SVZ progenitors

Norrin is a secreted molecule that can activate canonical Wnt signaling via Frizzled-4 and has been shown to also inhibit TGFβ signaling [45], [46]. While Wnt signaling has multiple roles during development, it has been shown to be critical for adult neurogenesis, and has been shown to be impaired in neurospheres isolated from recently-deceased AD patients [47]. As we find decreased NDP expression in the hippocampus of both late-onset AD brains and NDAD brains (Fig 6), suggesting a role for NDP in adult neurogenesis, we therefore tested recombinant norrin on primary murine adult subventricular zone (SVZ) progenitors as well as human iPSC-derived NPCs for effects of on proliferation. iPSC-derived NPCs (d14) did not show differences in proliferation in response to norrin (ki67 staining, data not shown), in line with the observation PS1 cells do not show greater proliferation at this time point of differentiation despite greater endogenous levels of NDP expression (ki67 staining, Fig 1G). However, adult SVZ progenitors from eight-week old mice did show increased proliferation in response to recombinant norrin, as measured by primary neurospehere formation, even in the presence of the mitogens EGF and FGF (p = 0.04; Fig S5). This supports a potential pro-neurogenic role for NDP in adult neurogenesis.

## Discussion

Human NPCs are an important cell type to study in context of AD, due to deficits in adult neurogenesis and newborn neuron survival seen in AD mouse models and potentially human patients [14]–[16], [47]. There might also be developmental components of FAD that could be reflected in NPCs. In addition, NPCs are at least somewhat more homogenous than the wide variety of neurons produced by current general neuronal differentiation protocols, which might allow better cross comparisons between control and FAD cells. To date, no study has addressed human iPSC-derived NPCs in the context of Alzheimer's disease and thus, we developed a *PSEN1* iPSC model that we used to interrogate potential alterations in FAD NPCs.

We first addressed potential differences in Aβ processing, the most critical component of known FAD pathology. Similar to a recent study of transdifferentiated *PSEN1* neurons [16], *PSEN1* NPCs have been shown to have higher Aβ42/Aβ40 ratios than equivalent control cells and in a greater magnitude than as fibroblasts (Fig 3). This is the first study of human NPCs that addresses pathogenic proteolytic processing of endogenous APP. Human NPCs therefore produce their own elevated supply of Aβ42:40 that may affect their developmental potential and survival.

Having established the validity of our FAD iPSC model, we then investigated whether we could identify novel molecules potentially important for AD. Towards this end, we performed molecular profiling experiments utilizing both undifferentiated and 14-day differentiated iPSCs from our core set (Fig 4). This led to the identification of 14 genes with altered expression in *PSEN1* NPCs (Table S2). Several of these also showed significant differences in late onset brains by either qPCR or data mining methods (Fig 6). Although we were not able to obtain FAD brain material to test our hits, our hope is that researchers with access to that tissue will examine our target genes in that context. While the differential gene expression changes identified in this manuscript are intriguing, they are only correlative at this point. It will require future mechanistic work in both human cells and animal models to determine whether they indeed play a functional role in Alzheimer's disease.

We looked at three targets in further detail: *NLRP2*, *ASB9*, and *NDP*. *NLRP2* was also the only gene differentially regulated in undifferentiated iPSCs. Control line 6842A was an outlier regarding *NLRP2* expression. Interestingly, the gene is located at the breakpoint of the balanced location in this line. It is likely that this chromosomal alteration is responsible for *NLRP2* expression level differences, although future studies will need to confirm this is the case. If true, such misregulation would be expected to have consequences for this balanced translocation. It is intriguing that *NLRP2* expression was decreased in late onset AD brains. One possibility is that the cells that express *NLRP2* at high levels (and thus would be predicted to have an increased pro-inflammatory response) might be more vulnerable in AD. On the other hand, *ASB9*, and E3-ligase (which inhibits mitochondrial function; [39]) was shown to be upregulated in brains from some late onset AD patients.

*NDP* is a particularly interesting target in the context of the proposal that FAD might have developmental consequences, in that 30% of patients with Norrie Disease, a disorder caused by mutation of *NDP*, have CNS deficits [45]. In addition, promotion of Wnt signaling and inhibition of TGFβ signaling by norrin should enhance adult neurogenesis by driving proliferation/NeuroD1 expression, and inhibiting astrocyte fate respectively [48], [49]. Thus the reduction of NDP in the hippocampus of late onset AD brains would be predicted to decrease neurogenic potential. Future studies incorporating gain and loss of function of NDP should help clarify NDP's potential role in neurogenesis and Alzheimer disease.

## Materials and Methods

See Figure S6 for extended material and methods.

### Cell Lines

Fibroblast 11 and 11C have been previously published [19]. All other fibroblasts were obtained from the Coriell Institute (Camden, NJ), and were reprogrammed to iPSCs in this study (see Figure S6 for details).

### Molecular Biology

Genomic DNA was prepared using the DNA Mini Kit, and RNA with the RNeasy Mini Kit, as per the manufacturer's instructions (Qiagen). qPCR was carried out on a Stratagene MX3000P QPCR machine (Agilent technologies) utilizing 40 cycles. cRNA for was amplified using the Illumina TotalPrep RNA Amplification Kit (Ambion) and ran on an Illumina HT_12_v4 BeadChip Array (Ilumina), as per the manufacturer's instructions. Oligos are found in Fig S6.

### Protein Analysis

Immunostaining was performed as has been described before [50]. Hoescht 33342 (Sigma) were used to visualize DNA. The following antibodies were used: OCT4 (Stemgent), SSEA4, Nanog (R&D Systems), Tra-160, Ki67, MAP2, Nestin, NeuN (Millipore), Tuj1 (Covance), NLRP2 (Santa Cruz), and NDP (Abnova). Quantification of immunostaining was done on the Celigo 200-BFFL Machine (Brooks Automation).

### Aβ Assays

Fibroblasts were split at 100,000 cells/6 well-well, and allowed to condition for 3 days before collection of conditioned media for analysis. Neuronal cultures were also conditioned for 3 days prior to collection of conditioned media at day 14. To quantify Aβ levels, human/rat Aβ 1–40 and 1–42 ELISA kits (Wako) were used according to the manufacturer's instructions. Duplicate assays were averaged for each biological replicate. There were 3 biological replicates for each line in each experiment (three independent-experiments per cell type). Assays were performed blindly.

### Human Brain Tissue Analysis

De-identified fresh frozen human autopsy brain tissue was obtained from the New York Brain Bank at Columbia University Medical Center (New York, NY). Neuropathological examination was per standardized protocols [51], [52].

## Supporting Information

Figure S1 **Related to Figure 1: Additional characterization of 7768C and neuronal differentiation.**
(PDF)

Figure S2 **Related to Table 1: Characterization of Core Lines**.
(PDF)

Figure S3 **Related to Figure 3: DapT Blocks total Aβ production.**
(PDF)

Figure S4 **Related to Figure 6: NDP Protein Is Expressed in Late-Onset AD Brains.**
(PDF)

Figure S5 **Related to Figure 6: Recombinant Norrin protein induces proliferation in adult SVZ neural progenitor cells (NPCs).**
(PDF)

Figure S6 **Extended material and Methods.**
(PDF)

Table S1 **Related to Figure 1: GO Terms associated with differentially regulated genes as determined by DAVID Functional Annotation.**
(DOCX)

Table S2 **qPCR Validation of Gene Expression Profile hits.**
(DOCX)
