# Supplementary material for: Characterization and Molecular Profiling of PSEN1 Familial Alzheimer's Disease iPSC-Derived Neural Progenitors
Source: PLoS One. 2014 Jan 8;9(1):e84547. doi: 10.1371/journal.pone.0084547 (PMC3885572; doi:10.1371/journal.pone.0084547)
Supplement: Figure S1 — Related to Figure 1: Additional characterization of 7768C and neuronal differentiation. (PDF) [file pone.0084547.s001.pdf]

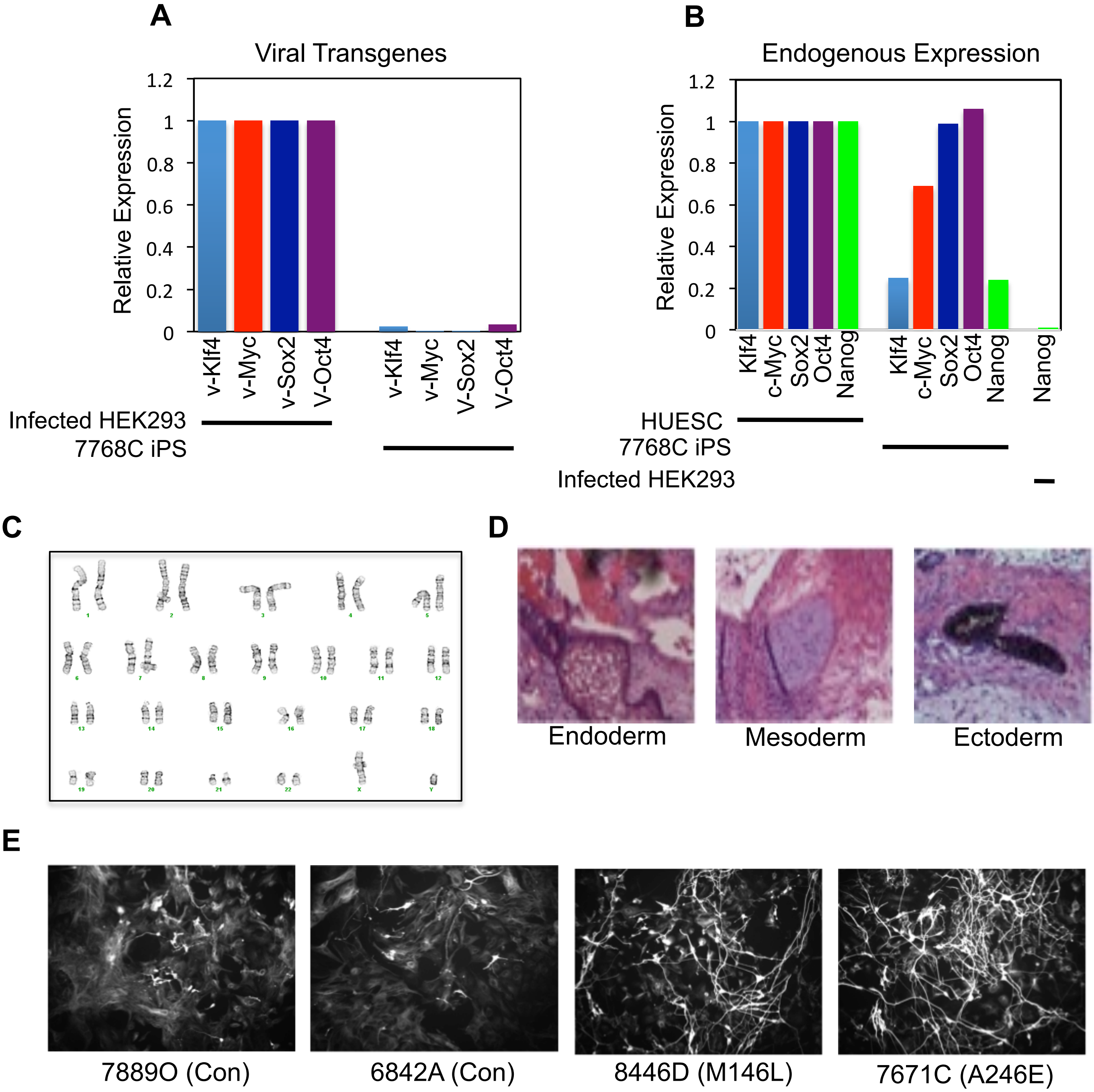

Figure S1, Related to Figure 1: Additional characterization of 7768C and neuronal differentiation. *A*. Real-time (RT) PCR of viral-specific transgene markers using RNA from virally infected 293 cells as a positive control. *B*. RT-PCR of endogenous stem cell genes, using RNA from human embryonic stem cells as a positive control, and virally infected HEK293 as a negative control. Beta-2 microglobulin expression was used to normalize all data for *A* and *B*. *C*. 7768C demonstrates a normal female karyotype as measured by G-banding (Cell Line Genetics). *D*. *In vivo* pluripotency was assessed by injection of undifferentiated 7768C iPSCs into NSG mice and harvesting the resulting teratoma for analysis. Paraffin-embedded sections were H&E stained and demonstrate cellular architecture consistent with all three germ layers. *E.* Day 14 differentiated cells produced patches of Tuj1+ neurons with complex morphology, which was more prevalent in *PSEN1* cells. Representative images of Tuj1 staining from 4 core cell lines.
