# Supplementary material for: Characterization and Molecular Profiling of PSEN1 Familial Alzheimer's Disease iPSC-Derived Neural Progenitors
Source: PLoS One. 2014 Jan 8;9(1):e84547. doi: 10.1371/journal.pone.0084547 (PMC3885572; doi:10.1371/journal.pone.0084547)
Supplement: Figure S2 — Related to Table 1: Characterization of Core Lines. (PDF) [file pone.0084547.s002.pdf]

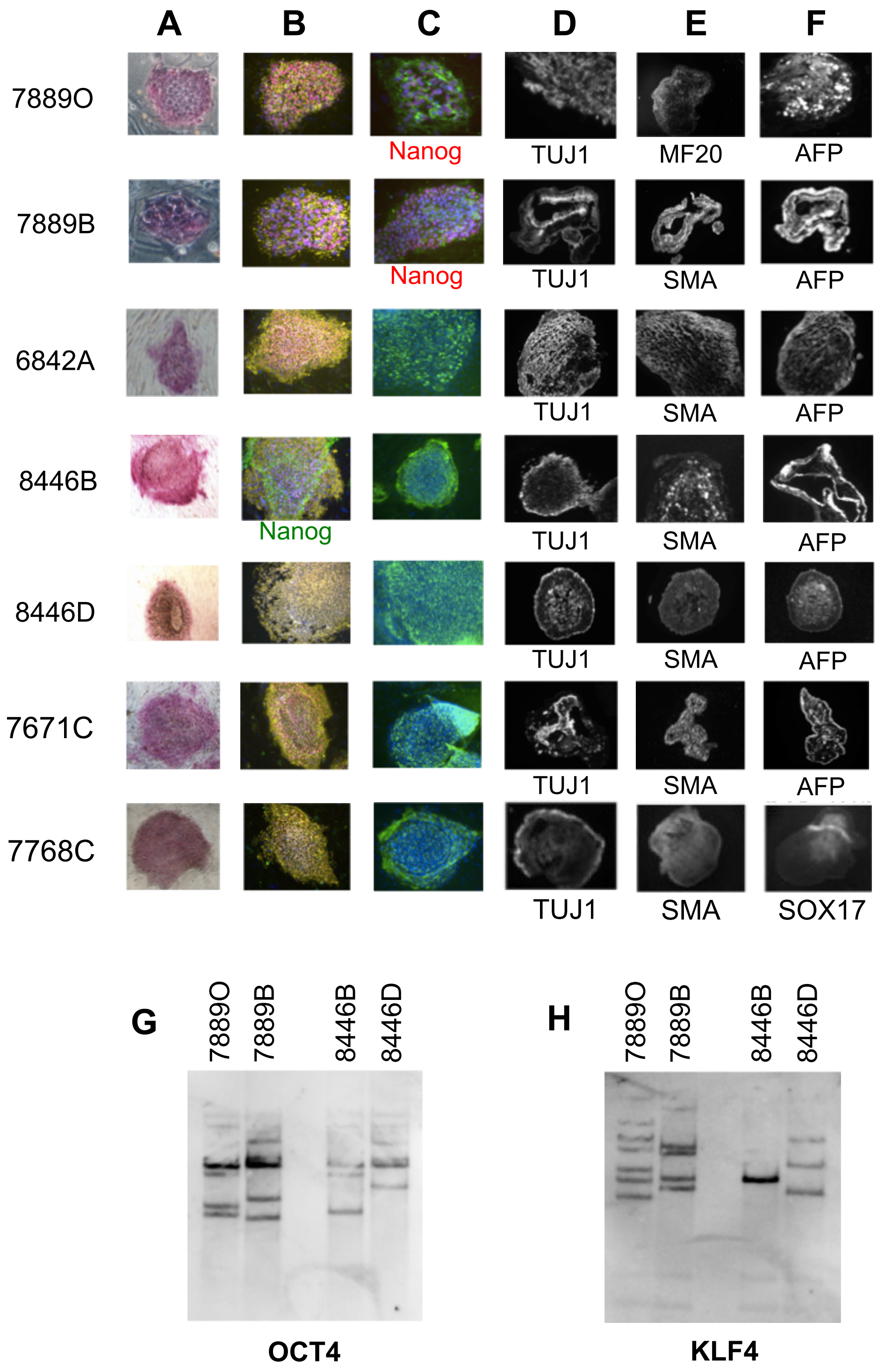

Figure S2, Related to Table 1: Characterization of Core Lines. *A*. Alkaline-Phosphatase activity assay. *B-C*. Immunofluorescence for relevant stem cell markers. DNA is in blue, Nanog is stained in panels as indicated. *B*, SSEA4 is in yellow, Oct4 is in red. *C*, Tra160 is in green. *D-F*. Immunofluorescence for three germ layers formed by Embryoid Bodies (EBs): *D*, Ectoderm, *E*, Mesoderm, *F*, Endoderm. *G-H*, Southern blotting using probes for either OCT4 or KLF4 demonstrate two independent iPSC clones derived from fibroblast 7889 (O and B) and 8446 (B and D).
