# Supplementary material for: Characterization and Molecular Profiling of PSEN1 Familial Alzheimer's Disease iPSC-Derived Neural Progenitors
Source: PLoS One. 2014 Jan 8;9(1):e84547. doi: 10.1371/journal.pone.0084547 (PMC3885572; doi:10.1371/journal.pone.0084547)
Supplement: Figure S3 — Related to Figure 3: DapT Blocks total Aβ production. (PDF) [file pone.0084547.s003.pdf]

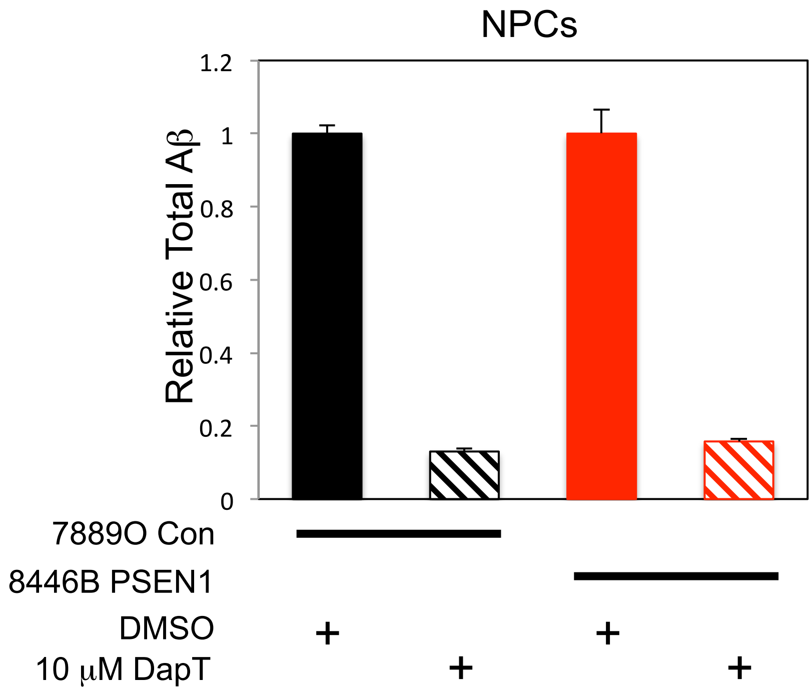

Figure S3, Related to Figure 3: DapT Blocks total Aβ production. Control and *PSEN1* NPCs/early neurons at day 13 of differentiation were treated with either DMSO vehicle or 10 μM DapT (triplicate biological replicates) for 24 hours. Conditioned media was then collected and analyzed for total Aβ (Aβ40 + Aβ42**)**. Both control and PSEN1 cells had equivalent levels of inhibition at this doseage of gamma-secretase inhibitor. One of two independent experiments with similar results is shown. N=3 biological replicates for each condition and error bars represent SEM.
