# Supplementary material for: Characterization and Molecular Profiling of PSEN1 Familial Alzheimer's Disease iPSC-Derived Neural Progenitors
Source: PLoS One. 2014 Jan 8;9(1):e84547. doi: 10.1371/journal.pone.0084547 (PMC3885572; doi:10.1371/journal.pone.0084547)
Supplement: Figure S4 — Related to Figure 6: NDP Protein Is Expressed in Late-Onset AD Brains. (PDF) [file pone.0084547.s004.pdf]

A

#	Status	Sex	Age	CERAD	Braak
1	Control	W	92	n.a	4
2	Control	M	74	A	2
3	Control	M	80	A	2
4	Control	W	77	A	3
5	Control	W	82	n.a.	3
6	AD	M	83	B	6
7	AD	W	85	B	5
8	AD	M	84	B	5
9	AD	M	83	B	5
10	AD	M	74	C	6
11	AD	M	82	C	6
12	AD	W	92	B	5
13	AD	M	92	B	5
14	AD	M	86	C	5
15	AD	W	89	C	6
16	AD	M	86	C	6

B

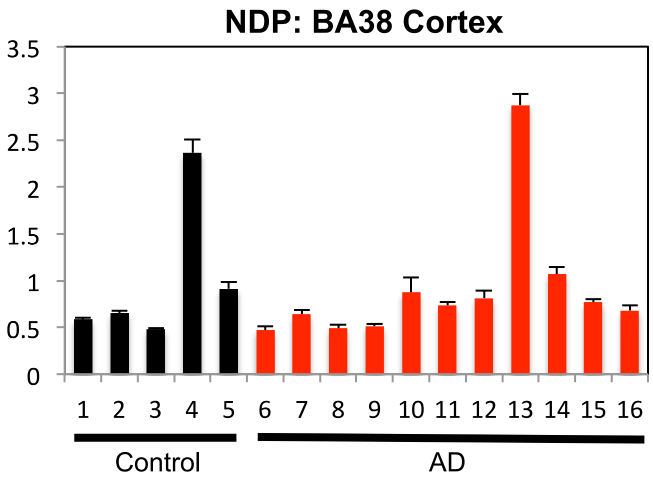

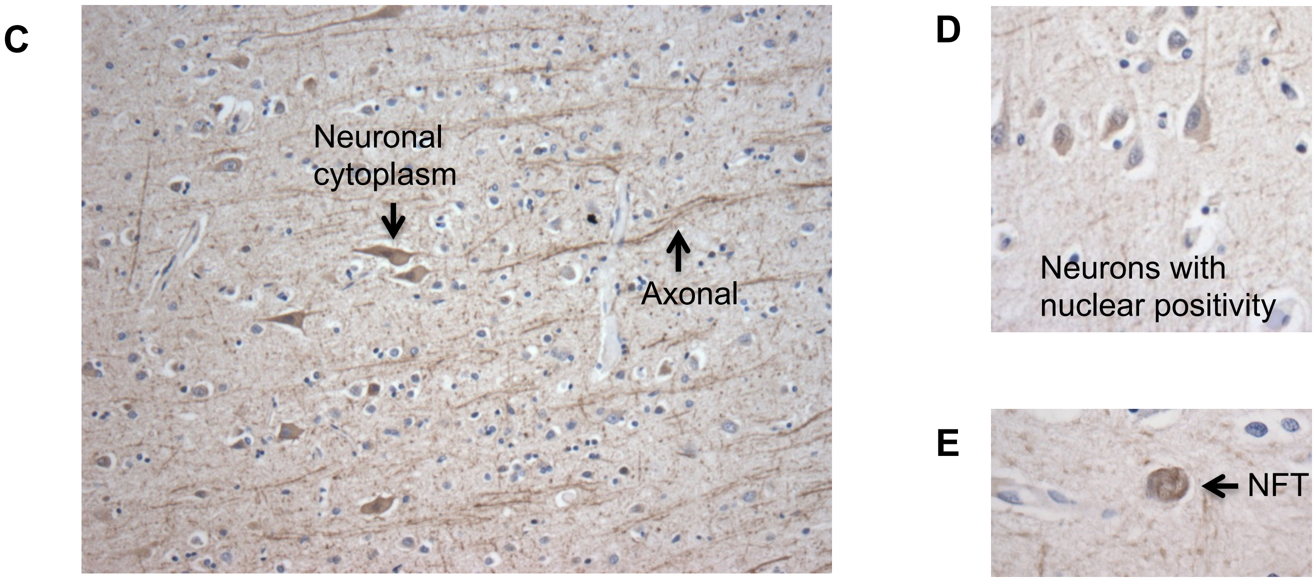

Figure S4, Related to Figure 6: NDP Protein Is Expressed in Late-Onset AD Brains. *A*. Description of samples utilized for Fig 6A,B and panel B. CERAD and Braak staging refer to amyloid load and extent of neurofibrillary tangles respectively. *B*. qPCR of *NDP* expression in area BA38 of control and AD patients as in Fig 6. Values are the average of technical replicates (3; error bars represent SEM) normalized to *GAPDH* and the average value of the 5 control lines. Black bars (1-5) are controls and red bars represent AD patients (6-16). *C-E*, Expression of *NDP* in a Late-Onset AD Brain, showing NDP DAB reactivity in neuronal cytoplasm and axons (*C*), neuronal nuclei (*D*) and some neurofibrillary tangles (*E*).
