# Supplementary material for: Characterization and Molecular Profiling of PSEN1 Familial Alzheimer's Disease iPSC-Derived Neural Progenitors
Source: PLoS One. 2014 Jan 8;9(1):e84547. doi: 10.1371/journal.pone.0084547 (PMC3885572; doi:10.1371/journal.pone.0084547)
Supplement: Figure S5 — Related to Figure 6: Recombinant Norrin protein induces proliferation in adult SVZ neural progenitor cells (NPCs). (PDF) [file pone.0084547.s005.pdf]

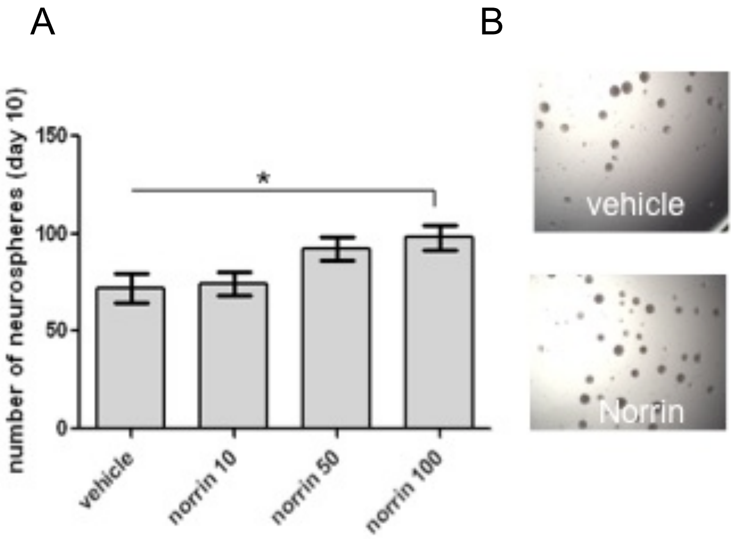

Figure S5, Related to Figure 5: **Recombinant Norrin protein induces proliferation in adult SVZ neural progenitor cells (NPCs).** *A*. The number of neurospheres formed after 10 *days in vitro* in which NPCs were treated with 10, 50 or 100ng/mL recombinant Norrin protein, or vehicle control. *B*. Representative images of neurosphere formation assay showing the effect of recombinant Norrin protein (100ng/ml) on the treated NPCs. Error bars represent standard error of the mean. *P < 0.05, analysis of variance with Dunnet's *post hoc* analysis.
