# Supplementary material for: Characterization and Molecular Profiling of PSEN1 Familial Alzheimer's Disease iPSC-Derived Neural Progenitors
Source: PLoS One. 2014 Jan 8;9(1):e84547. doi: 10.1371/journal.pone.0084547 (PMC3885572; doi:10.1371/journal.pone.0084547)
Supplement: Figure S6 — Extended material and Methods. (PDF) [file pone.0084547.s006.pdf]

Figure S6: Extended material and Methods

Retroviral Reprogramming: Fibroblasts were plated at 30,000 – 50,000 cells in a single well of a 6-well plate or multiple wells of a 12- well-plate, which was infected the 6-18 hours later with four retroviruses prepared for Oct4, KLF4, Sox2, and c-Myc by the Harvard Gene Therapy Core. Infected fibroblasts were split 7-14 days post-infection onto MEFs (γ-irradiated mouse embryonic fibroblasts) and concomitantly treated with three chemical compounds to enhance reprogramming: SB431512 (2 μm), Thiazovivan (0.5 μM), and PD0325901 (0.5 μM)(Lin et al., 2009).

Cell culture: Cell culture reagents were from Invitrogen unless otherwise stated, and all media contained penicillin-streptomycin (100U/mL-0.1mg/mL). Human fibroblasts were maintained on TC plates coated with 0.1% gelatin and grown in FM10 Media (DMEM/10%FBS/Glutamax (2mM)/2-Mercaptoethanol (0.1mM). Cells were initially quarantined until they were tested for the absence of mycoplasma. Undifferentiated iPSCs were kept on irradiated MEFs (Globalstem) plated on TC plates with 0.1% gelatin, and grown in HUESM (20% KSR/KO-DMEM/Non-essential amino acids (0.1mM)/Glutamax (2mM) 2-Mercaptoethanol (0.1mM)/bFGF (10ng/ml)). Monolayer neuronal differentiation was carried out in custom mTesR1 (minus 5 growth factors) and Neurobasal media supplemented with B-27 minus retinoic acid. PD0325901, SB431542, Thiazovivin, and LDN-193189 were from Stemgent. Matrigel, natural mouse laminin and polyornithine were also from Invitrogen. Karyotyping and fingerprinting of cell lines was done by Cell Line Genetics (Madison, WI).

Monolayer Neuronal Differentiation: Briefly, iPSC colonies grown on MEFs were cleaned to remove differentiated cells and pre-plated on gelatin-coated plates to remove MEFs. The resulting SNF was spun and resuspended in mTesR1 media containing 10 μM ROCK inhibitor (Y-27632, Stemgent), plated at a density of 200,000 cells per 6 well-well on polyornithine (100μg/mL)/laminin (3 μg/mL), and allowed to recover for 3 days to allow near confluency. Cells

were neuronally differentiated with dual-smad inhibition from days 0-9 in custom TesR1 (5x supplement, 1/100 Pen-Strep) using 10μM SB431542 and 250 nM LDN193189. Cell were split with acutase on Day 9, and plated in a similar density and substrate as the initial plating. Media was changed from custom mTESR1 in Neurobasal + B27 supplement` (no retinoic acid) in a stepwise fashion, and cells were fed every 2 to 3 days until analyzed.

Flow Cytometry for CD56: Cells were enzymatically harvested with Accutase above to obtain a single cell suspension prior to resuspension in 100 μl of a sterile iPSC staining buffer [DPBS containing 0.5% bovine serum albumin fraction V (BSA; Invitrogen), 100 U/ml penicillin/streptomycin (Invitrogen), 2 mM EDTA (Invitrogen), and 20 mM glucose (Sigma)]. Fluorescence-conjugated CD56 (1 μl anti-CD56, catalog #s: 560360, BD Biosciences) were added to cells that had been filtered through a 35 um cell strainer (BD Biosciences). Cells were incubated at room temperature (RT) for 20 minutes shielded from light. The stained cells were washed once with iPSC staining buffer and were analyzed immediately on a 5 laser BD Biosciences ARIA-IIu™ SOU Cell Sorter configured with a 100 μm ceramic nozzle and operating at 20 psi sheath fluid pressure.

Electrophysiology: Neurons were cultured on polyornithine and laminin coated coverslips and recorded using whole cell patch between day 35 and 55. The recording bath solution consisted of (in mM): NaCl (119), KCl (5), HEPES (20), Glucose (30), $MgCl_2$ (2), $CaCl_2$ (2), glycine (0.001), and picrotoxin (0.1). Glass electrodes with a resistance between 5.5-7 MΩ, were filled with an intracellular solution containing (in mM): K-Gluconate (130), KCl (10), HEPES (5), $CaCl_2$ (0.06), $MgCl_2$ (5), EGTA (0.6), ATP (2), GTP (0.2), Phosphocreatine (20) and Leupeptine (0.2) and 50 U/ml creatine-phosphokinase. Recordings were made with a Warner amplifier (model PC-501A) and filtered at 1 kHz. Signals were sampled at 10 kHz with 16-bit resolution with a Digidata 1440A (Axon Instrument), and analysed with Matlab 8.0 (MathWorks). To evoke action-potentials, current clamp steps were given (0 pA for 100 ms, steps from -60 pA to +120 pA, 20 pA each, for 1 s). Action potentials were subsequently blocked with perfusion of 1 μM TTX. Na^{2+}

and K^+- currents were obtained in voltage clamp mode (holding potential: -70 mV for 100 ms, steps from -90 mV to +20 mV, 10 mV each, for 1 s). Sodium and potassium currents were blocked through 1 μM TTX and 10 mM TEA, respectively.

Calcium Studies: iPSC line 7889O neuronal cultures that had been neuronally differentiated for 45 days were pretreated with an HBSS loading buffer containing 20 mM HEPES, 2.5 mM probenecid, and Fluo-4NW (Invitrogen) for 30 min at 37°C and then at room temperature for an additional 30 min before imaging, following the manufacturer's instructions. Cells were then placed in an imaging chamber and imaged on a Zeiss LSM 510 inverted confocal microscope using a 20X objective (0.8 n.a.). Time-lapse images were taken every 1.5 seconds for 3 minutes using 512 X 512 pixel resolution and cells were excited using the 488 nm laser line. 4 uM X 4 uM ROIs were drawn over each cell soma and change in fluorescence intensity over time was calculated within the imaging software. After background subtraction, Ca2+ signals were then analyzed by hand and with a custom written routine in Matlab (Mathworks, Natick, MA) based on the “peakfinder” and “relevantpeaks” functions in Matlab. Observers were blind to each condition.

Recombinant Norrin Treatment of SVZ Progenitors: Adult FVB/N mice were killed by cervical dislocation (n=3 per preparation) and their brains removed and placed into Pipes buffer (20mM Pipes, 25mM glucose, 0.12M NaCl , 0.5 mM KCl [ph 7.4]). The lateral walls of the lateral ventricles were dissected, collected in Pipes buffer and incubated in a mixture containing 1.2mL of activated papain and 8.8 ml of Pipes buffer. Papain was activated 20min in advance by placing 3 mg of papain (Worthington Biochemical) in 1 ml of 1.1 mM EDTA, 5.5 mM cysteine HCl. The pieces of tissue were incubated in papain solution at 37ºC on a rocking platform for 10min and collected by centrifugation at 1300rpm for 5 min. The cellular pellet was resuspended in 1 ml of DMEM-F12 (GIBCO) containing 0.7 mg of ovomucoid inhibitor and 1 mg of DNase. The cells were dissociated and neural precursor cells were separated from differentiated cells, myelin

and extracellular matrix using density gradient separation by centrifugation step with 22% Percoll. Pelleted cells were separated from supernatant, washed and resuspended in 1ml neurosphere proliferation media (DMEM-F12 (GIBCO) supplemented with N2 (GIBCO), 2mM glutamine, 0.6% (w/v glucose), insulin-transferrin-selenium (GIBCO), and 15 mM HEPES, B27, penicillin/streptomycin). The cells were counted and plated in 96well-low attachment plate (Corning) (1.5 x10^5 /mL) in the same medium containing 10ng/mL of EGF receptor grade (Millipore) and 10ng/mL human bFGF (Invitrogen). Two days after isolation neurospheres were grown in the presence of different concentrations (10, 50 and 100ng/mL) of the recombinant Norrin protein (R&D systems), media, or vehicle control (Norrin was resuspended in 4mM HCL/0.1% BSA). The numbers of neurospheres were counted for six 40x fields /well after 10 days in vitro. Each experiment was performed in triplicate for each condition and a total of three independent experiments were included.

Analysis of Laser Captured Microdissected (LCM) Neuron GEP Data:

Analysis was performed on a GEP dataset (GSE5281) downloaded from the Gene Expression Omnibus (GEO) database. The CEL files were downloaded and analyzed using BRB ArrayTools (available at http://linus.nci.nih.gov/BRB-ArrayTools.html). The CEL files were normalized and filtered prior to comparing gene expression levels. First, the downloaded CEL files were normalized using the MAS5 algorithm. Next, probesets that had the lowest variance across the samples were filtered out. The significance of the variance was calculated for each probeset by comparing the variance of their log-expression values to the median of all the variances. Statistical signifiance was measured using the corrected FDR value and probesets that had a variance of less than the FDR-corrected p-value < 0.1 were filtered out. Out of 54,000 total probesets on the array, 17,224 probesets passed this filter. Comparison of gene expression levels was performed on the normalized and filtered dataset and significance was measured with the FDR-corrected p-value. The following are the number of individuals

analyzed per brain region: Hippocampus: Control 13, NDAD 6, AD 10; Entorhinal Cortex: Control 13, NDAD 6; Middle Temporal Gyrus: Control 12, NDAD 6, AD 16

Oligos:

QPCR Primers

Oct 4 (endogenous) F: CCCCAGGGCCCCATTTTGGTACC R: GGCACAAACTCCAGGTTTTC

Sox2 (endogenous) F: ACACTGCCCCTCTCACACAT R: GGGTTTTCTCCATGCTGTTTCT

Klf4 (endogenous) F: ACCCACACAGGTGAGAAACCTT R: GTTGGGAACTTGACCATGATTG

c-Myc (endogenous) F: AGCAGAGGAGCAAAAGCTCATT R:CCAAAGTCCAATTTGAGGCAGT

Oct4 (transgene) F: CCCCAGGGCCCCATTTTGGTACC R: AACCTACAGGTGGGGTCTTTCA

Sox2 (transgene) F: ACACTGCCCCTCTCACACAT R: AACCTACAGGTGGGGTCTTTCA

Klf4 (transgene) F: GACCACCTCGCCTTACACAT R: AACCTACAGGTGGGGTCTTTCA

c-Myc (transgene) F: AGCAGAGGAGCAAAAGCTCATT R: AACCTACAGGTGGGGTCTTTCA

B2M F: TAGCTGTGCTCGGGCTACT R: TCTCTGCTGGATGACGCG

GAPDH F: ACATCGCTCAGACACCATG R: TGTAGTTGAGGTCAATGAAGGG

ABCC2 F: AGGAGATTTGGCTGAGATTGG R: TTTCCTACATGAGCATCCACTG

ADM2 F: GACCCGTCAAACCCAGG R: GAGGCTGACCCATAACAGG

ASB9 F: ACGGCAGATCATGTTTCCC R: CACAAGCATTAAACAGTGGAGTG

BIK F: TCTTTGGAATGCATGGAGGG R: GTAGATGAAAGCCAGACCCAG

C7orf16 F: TCTTCATAACACTGACCTGGAAC R: ACCATCCTTTTCGTCATCTTCC

ECEL1 F: CCCTCTTTGTACATGAGCACT R: ACTTGATGTCTTCCACTAGCTG

EGFL8 F: AACCAGTGCCAGCATACTC R: CTCGTGAATCTCCTGCTTCAG

FLJ35024 F: CAAACAAGAGGTCCCTGGATC R: AGTTGATGCCTGGTCTGAAG

FSTL5 F: GATCAGGTCTGGGTGCTAAG R: CCACTCTGTCAAATTGCTTTCC

MT2A F: GGCTCCTGCAAATGCAAA R: CAGCAGCTGCACTTGTCC

NDP F: GCGTCACGCTCCGAGCCTTT R: CTGAGCATCGCAGCCGCAGT

NLRP2: CTTGGTGCTTTGGAACTGCGACAT R: TTGCACAGTGGTTTCCTCAAAGCC

PLP1 F: CTGGCTGAGGGCTTCTACAC R: CCTAGCCATTTTCCCAAACA

PTGS2: F: ACAGGCTTCCATTGACCAG R: TCACCATAGAGTGCTTCCAAC

SLC45A F: AGACCCTACATCCTCACCC R: AAAGAGAACGACACCTATCATGG

SMOC1 F: TGGTTCAGGCCATTAACTCAG R: TCTCCCGCTTGTTAATGTCG

TBX2 F: AGTTCCACAAGCTAGGCAC R: ACTTATAGCGGCAATCGTCAG

TUBB4A: F: TGCAACTGGAGAGGATCAAC R: CCGAAAGATCTGACCGAAGG

ZNF300 F: CAGTTTCCAAACCAGATGTCATC R: ACTGGGAGTTGTGAAGGTTAC

Southern Blot Probes

Oct4 F: GAGAAGGAGAAGCTGGAGCA R: GTGAAGTGAGGGCTCCCATA

Klf4 F: ACCTGGCGAGTCTGACATGG R: TCTTCATGTGTAAGGCGAGGTGG
