# Supplementary material for: Characterization and Molecular Profiling of PSEN1 Familial Alzheimer's Disease iPSC-Derived Neural Progenitors
Source: PLoS One. 2014 Jan 8;9(1):e84547. doi: 10.1371/journal.pone.0084547 (PMC3885572; doi:10.1371/journal.pone.0084547)
Supplement: Table S1 — Related to Figure 1: GO Terms associated with differentially regulated genes as determined by DAVID Functional Annotation. (DOCX) [file pone.0084547.s007.docx]

| **ASSOCIATED GO TERMS FOR UPREGULATED GENES IN *PSEN1* NPCs** | **# Genes** | **pVALUE** |
| --- | --- | --- |
| GO:0044421~extracellular region part | 19 | 0.008 |
| GO:0005576~extracellular region | 31 | 0.017 |
| GO:0016481~negative regulation of transcription | 11 | 0.018 |
| GO:0010629~negative regulation of gene expression | 11 | 0.032 |
| GO:0006357~regulation of transcription from RNA polymerase II promoter | 14 | 0.033 |
| GO:0045934~negative regulation of nucleobase, nucleoside, nucleotide and nucleic acid metabolic process | 11 | 0.035 |
| GO:0000166~nucleotide binding | 32 | 0.038 |
| GO:0051172~negative regulation of nitrogen compound metabolic process | 11 | 0.038 |
| GO:0043565~sequence-specific DNA binding | 12 | 0.043 |

| **ASSOCIATED GO TERMS FOR DOWNREGULATED GENES IN *PSEN1* NPCs** | **# Genes** | **pVALUE** |
| --- | --- | --- |
| GO:0051270~regulation of cell motion | 10 | 7.60E-06 |
| GO:0010033~response to organic substance | 17 | 3.34E-05 |
| GO:0043549~regulation of kinase activity | 12 | 3.61E-05 |
| GO:0051338~regulation of transferase activity | 12 | 5.24E-05 |
| GO:0042325~regulation of phosphorylation | 13 | 8.91E-05 |
| GO:0009719~response to endogenous stimulus | 12 | 1.12E-04 |
| GO:0051174~regulation of phosphorus metabolic process | 13 | 1.30E-04 |
| GO:0019220~regulation of phosphate metabolic process | 13 | 1.30E-04 |
| GO:0045859~regulation of protein kinase activity | 11 | 1.38E-04 |
| GO:0009725~response to hormone stimulus | 11 | 2.28E-04 |
| GO:0042981~regulation of apoptosis | 16 | 4.06E-04 |
| GO:0043067~regulation of programmed cell death | 16 | 4.51E-04 |
| GO:0010941~regulation of cell death | 16 | 4.69E-04 |
| GO:0042127~regulation of cell proliferation | 14 | 0.003 |
| GO:0005576~extracellular region | 24 | 0.020 |
| GO:0031328~positive regulation of cellular biosynthetic process | 11 | 0.020 |
| GO:0009891~positive regulation of biosynthetic process | 11 | 0.022 |
| GO:0006915~apoptosis | 10 | 0.024 |
| GO:0012501~programmed cell death | 10 | 0.026 |
| GO:0005886~plasma membrane | 38 | 0.031 |

**Table S1, Related to Figure 4: GO Terms associated with differentially regulated genes as determined by DAVID Functional Annotation.**
