# Supplementary material for: Characterization and Molecular Profiling of PSEN1 Familial Alzheimer's Disease iPSC-Derived Neural Progenitors
Source: PLoS One. 2014 Jan 8;9(1):e84547. doi: 10.1371/journal.pone.0084547 (PMC3885572; doi:10.1371/journal.pone.0084547)
Supplement: Table S2 — qPCR Validation of Gene Expression Profile hits. (DOCX) [file pone.0084547.s008.docx]

|  | **7889O** | **7889B** | **11C** | **6842A** | **8446B** | **8446D** | **7671C** | **7768C** | **Con Avg** | **PS1 Avg** | **Ratio** | **t-Test** |
| --- | --- | --- | --- | --- | --- | --- | --- | --- | --- | --- | --- | --- |
| **Higher PS1** |  |  |  |  |  |  |  |  |  |  |  |  |
| ASB9 | 1 | 2.3 | 1.9 | 2.4 | 4.2 | 4.6 | 3.4 | 5.8 | 1.91 | 4.50 | 2.37 | 0.01 |
| BIK | 1 | 1.43 | 1.55 | 1.37 | 2.97 | 4.54 | 7.97 | 0.87 | 1.31 | 4.09 | 3.13 | 0.04 |
| C7orf16 | 1 | 4.72 | 5.73 | 4.70 | 18.48 | 18.44 | 23.12 | 2.52 | 4.04 | 15.64 | 3.88 | 0.03 |
| NDP | 1 | 1.42 | 3.47 | 1.74 | 4.01 | 7.09 | 14.01 | 8.96 | 1.91 | 8.52 | 4.46 | 0.01 |
| NLRP2 | 1 | 4.8 | 6.3 | 42.5 | 48.7 | 53.8 | 43.9 | 70.2 | 20.99 | 54.16 | 2.58 | 0.001 |
| PLP1 | 1 | 1.45 | 2.69 | 3.04 | 4.46 | 9.42 | 13.73 | 8.13 | 2.04 | 8.93 | 4.37 | 0.02 |
| SLC45A2 | 1 | 2.14 | 2.54 | 2.94 | 10.45 | 11.26 | 14.65 | 2.77 | 2.16 | 9.78 | 4.54 | 0.01 |
| TBX2 | 1 | 2.90 | 2.79 | 1.90 | 12.29 | 7.11 | 7.80 | 2.86 | 2.15 | 7.51 | 3.50 | 0.03 |
| TUBB4 | 1 | 1.92 | 2.66 | 2.37 | 8.20 | 14.71 | 19.08 | 1.32 | 1.99 | 10.83 | 5.45 | 0.02 |
| ZNF300 | 1 | 1.32 | 0.74 | 2.65 | 3.04 | 4.84 | 2.72 | 3.91 | 1.42 | 3.63 | 2.55 | 0.02 |
| **Lower PS1** |  |  |  |  |  |  |  |  |  |  |  |  |
| ADM2 | 1 | 0.23 | 0.86 | 0.49 | 0.21 | 0.53 | 0.16 | 0.28 | 0.64 | 0.29 | 0.46 | 0.03 |
| FLJ35024 | 1 | 0.38 | 2.06 | 0.16 | 0.06 | 0.08 | 0.13 | 0.48 | 0.90 | 0.18 | 0.20 | 0.04 |
| MT2A | 1 | 0.50 | 0.91 | 0.39 | 0.64 | 0.37 | 0.13 | 0.28 | 0.70 | 0.35 | 0.50 | 0.05 |
| PTGS2 | 1 | 0.91 | 1.89 | 0.22 | 0.41 | 0.30 | 0.16 | 0.26 | 1.00 | 0.28 | 0.28 | 0.02 |
| **Trend PS1** |  |  |  |  |  |  |  |  |  |  |  |  |
| ABCC2 | 1 | 8.66 | 6.28 | 6.24 | 24.52 | 31.75 | 71.36 | 1.90 | 8.78 | 34.24 | 3.90 | 0.07 |
| ECEL1 | 1 | 4.93 | 0.94 | 4.58 | 23.13 | 23.43 | 24.98 | 1.41 | 3.14 | 18.24 | 5.81 | 0.07 |
| EGFL8 | 1 | 1.14 | 1.44 | 1.26 | 2.26 | 1.96 | 2.56 | 0.95 | 1.21 | 1.93 | 1.60 | 0.06 |
| FSTL5 | 1 | 4.01 | 3.82 | 2.89 | 5.06 | 6.08 | 14.13 | 3.04 | 2.93 | 7.08 | 2.42 | 0.06 |
| SMOC1 | 1 | 1.08 | 1.70 | 1.07 | 3.80 | 6.05 | 14.49 | 0.78 | 1.21 | 6.28 | 5.19 | 0.08 |

**Table S2, Related to Figure 5:** **qPCR Validation of Gene Expression Profile hits**. Relative expression values for each cell line reflect the averages of 2 independent experiments with 3 biological replicates per experiment. Student’s t-Test reflects differences between control and *PSEN1* NPCs and was calculated using data points from two independent experiments rather than the average listed.
